# A Novel Ionospheric Disturbance Index to Evaluate the Global Effect on BeiDou Navigation Satellite System Signal Caused by the Moderate Geomagnetic Storm on May 12, 2021

**DOI:** 10.3390/s23031183

**Published:** 2023-01-20

**Authors:** Liming He, Cong Guo, Quanyou Yue, Shixuan Zhang, Zenghui Qin, Junfei Zhang

**Affiliations:** 1 School of Resources and Civil Engineering, Northeastern University, Shenyang 110819, China; 2 National Time Service Center, Chinese Academy of Sciences, Xi’an 710600, China

**Keywords:** BeiDou navigation satellite system, geomagnetic storm, ionosphere, signal quality, precise point positioning

## Abstract

In this paper, we propose a new method to quantitatively evaluate the quality of the carrier phase observation signals of the BeiDou Navigation Satellite System (BDS) during weak and moderate geomagnetic storms. We take a moderate geomagnetic storm that occurred on 12 May 2021 during the 25th solar cycle as an example. The results show that the newly defined PAS (Percentage of Affected Satellites) index shows significant anomaly changes during the moderate geomagnetic storm. Its variation trend has good correlations with the geomagnetic storm Kp index and Dst index. The anomaly stations are mainly distributed in the equatorial region and auroral region in the northern and southern hemispheres. The proposed PAS index has a good indication for both BDS2 and BDS3 satellites. We further validated this index by calculating the Precise Point Position (PPP) positioning error. We found that the anomaly period of PAS has strong consistency with the abnormal period of PPP positioning accuracy. This study could provide methodological support for the evaluation of the signal quality and analysis of positioning accuracy for the BeiDou satellite navigation system under different space weather conditions.

## 1. Introduction

The plasma ejected from the surface of the Sun propagates through interplanetary space to the top of the Earth’s magnetosphere, and the enormous energy radiated by it interacts with the Earth’s magnetic field resulting in geomagnetic storms [1,2]. As a common natural disaster phenomenon, geomagnetic storms can cause irregular changes in the total electron content (TEC) of the ionosphere [3] and certain scale irregularities result in ionospheric scintillations, that is, the amplitude and phase of radio signals passing through the ionosphere will change irregularly. Therefore, ionospheric scintillations are generally classified into amplitude scintillations and phase scintillations. The amplitude scintillations are mainly distributed in the magnetic equatorial regions of the Earth and the phase scintillations are more frequently distributed in the high latitudes of the Earth [4,5]. When ionospheric scintillation occurs, it can directly cause abnormal disturbances or even interruptions in the signal of the satellite navigation system, which seriously affects the signal quality of the satellite navigation system and the reliability of the positioning accuracy. It brings a disastrous impact on many fields that rely on satellite navigation and positioning technology [6,7].

Global Navigation Satellite Systems (GNSS) are space-based radio navigation and positioning systems providing users with all-weather three-dimensional coordinates, velocity, and time information anywhere on the Earth’s surface or in near-Earth space. To date, there are four main constellations in orbit including the USA’s Global Positioning System (GPS), Russia’s GLONASS, the European Union’s GALILEO, and China’s BeiDou Navigation Satellite System (BDS). Currently, GNSS are widely used in the military, transportation, agriculture, electric power, telecommunication, resource and environment, disaster prevention and mitigation, surveying and mapping, engineering construction, urban management, internet of things, location services fields, and other fields [8,9,10]. A large number of studies have been conducted to quantify the quality of GPS observation signals and monitor ionospheric disturbances during geomagnetic storms. The amplitude scintillation index S_4_ and the phase scintillation index $\sigma_{\emptyset }\;$are often used to assess the ionospheric scintillation intensity [11]. Pi et al. [12] first proposed the calculation method for the TEC rate of change and defined the Rate of TEC index (ROTI) in 1997. Afraimovich et al. [13,14] pioneered the concept of using “phase slip” to study the effects of geomagnetic storms on GPS signals. In addition, several improved indices based on ROTI and S_4_ have been proposed for ionospheric scintillation monitoring, such as the Along Arc TEC Rate (AATR) index [15], the ionosphere-free standard deviation (σ_IF_) [16], the disturbance ionosphere index spatial gradient (DIXSG) [17], and the S_4c_ index [18]. Yue et al. [19] found a correlation between the dense GPS signal loss phenomenon and the high fluctuation of ROTI. Dense loss of GPS signal generally refers to the phenomenon of a large number of satellite signals lost within a local region. By studying GPS scintillation, TEC loss, and ROTI, Deng et al. [20] found that the simultaneous occurrence of strong scintillation, TEC loss, and ROTI may be related to ionospheric irregularities. Xu et al. [21] used machine learning methods to predict ROTI at high-latitude sites in Canada. Wang et al. [22] analyzed the TEC, ROTI, and GPS positioning accuracy at high-latitude regions in the northern hemisphere during strong geomagnetic storms. Their results showed that there was a good agreement between the ionospheric phase scintillation index and ROTI index, and the positioning error of many stations increased significantly during the scintillation occurrence. Wu et al. [23] proposed a new global ROTI perturbation index (GROTI). They statistically analyzed the GROTI with geomagnetic indices Kp and AE at high, middle, and low latitudes, respectively, and the results show that there is a good correlation between the GROTI and geomagnetic indices Kp and AE in the high latitude region. He et al. [24,25] showed that the rate of global GPS signal loss during severe and great geomagnetic storms increased significantly compared with the reference days. The signal loss mainly occurred during the main phase and recovery phase of the geomagnetic storms, and the low-frequency L2 signal of GPS was more easily affected by the geomagnetic storms than the high-frequency L1 signal.

However, previous studies mainly focus on strong, severe, and great geomagnetic storms, and few studies on weak and moderate geomagnetic storms. Moreover, the evaluation of the signal quality of the single station is mainly based on the ROTI index, which will fail under the condition with lost satellite signals. The S_4_ index and $\sigma_{\emptyset }$ index can only be obtained using a dedicated and costly ionospheric scintillation monitoring receiver, which requires a high sampling rate. As a satellite navigation and positioning system independently developed and operated by China, BDS currently covers the whole world, providing global users with integrated services of positioning, navigation, timing, and communication (PNTC) [26,27]. Therefore, it is of great importance to evaluate the observation signal quality and the influence of positioning accuracy of BDS during frequently occurred weak and moderate geomagnetic storms. Taking a moderate geomagnetic storm that occurred on 12 May 2021 as an example, this paper uses the raw observation data of the global BDS continuously operating stations to perform a statistical analysis of the BDS observation signal and proposes a method that can quantitatively evaluate the observation signal quality of the global satellite navigation and positioning system during the weak and moderate geomagnetic storms, which can avoid the failure scenarios with lost satellite signals based on the use of common GNSS dual-frequency receivers. We further analyze and verify the PPP positioning error during the geomagnetic storm. This study provides a new method for quantitative evaluation of BDS observation signal quality, which is of great significance to guarantee the application of BDS in PNTC under different space weather conditions.

This paper is structured as follows. Chapter 1 introduces the background of this paper. Section 2 presents the selected geomagnetic storm event, data source, and data processing flow. The definition of GNSS observation signal quality evaluation index is described in Section 3. Section 4 presents the detailed results and analysis. In Section 5, we discuss the temporal correlation between the geomagnetic storm index and the proposed index, the spatial distribution characteristics of the affected stations, comparison between the PAS Index and the ROTI Index and the influence of geomagnetic storms on PPP. Finally, the conclusions are given in Section 6.

## 2. Geomagnetic Storm Event and Data Source

Geomagnetic indices are usually used to describe the overall level of geomagnetic activity, and the intensity of geomagnetic disturbances in a certain period observed by certain spatially distributed stations is used for classification. In this paper, we use geomagnetic indices Kp and Dst to describe the intensity of the geomagnetic storm. Kp index is a widely used geomagnetic index, which mainly reflects the global average geomagnetic activity intensity and takes a value every three hours, ranging from 0 to 9. The Kp index can be generally classified into 28 levels: 0, 0+, 1−, 1, 1+, …, 9−, 9. The Dst index can be used to characterize the complete process of a geomagnetic storm from its occurrence to its end. According to the classification criteria of Loewe and Prölss (1997), −50 nT < Dst ≤ −30 nT is a weak geomagnetic storm, −100 nT < Dst ≤ −50 nT is a moderate geomagnetic storm, −200 nT < Dst ≤ −100 nT is a strong geomagnetic storm, −350 nT< Dst ≤ −200 nT is a severe geomagnetic storm, and Dst ≤ −350 nT is a great geomagnetic storm. The stronger the geomagnetic storm, the more intense the geomagnetic disturbances and the greater the hazard caused by the geomagnetic storm [28].

**Figure 1:**
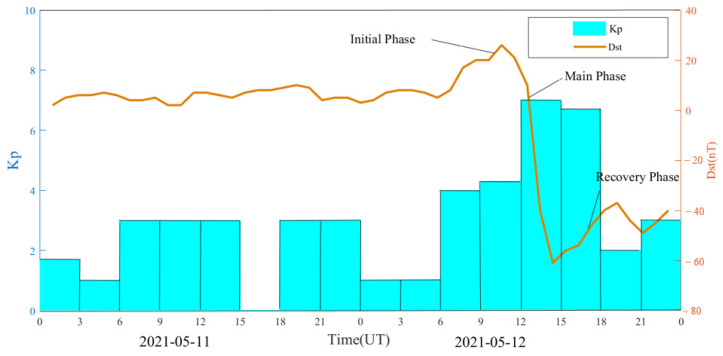
Kp and Dst indices from 11 to 12 May 2021.

The geomagnetic storm event on 12 May 2021 was the first moderate geomagnetic storm of the 25th solar cycle. The Kp index increased sharply from 12:00 UT to a maximum value of 7 and continued until 18:00 UT. The Dst index reached a minimum value of −61 nT at 15:00 UT. The Dst index remained below −30 nT from 14:00 UT until noon on 13 May, when it returned to a quiet level. In order to analyze the quality of BDS observation signal and PPP positioning error during the geomagnetic storm, it is necessary to select a reference day for comparison. For the selection of the reference day, the following principles are generally followed: (1) the change of the geomagnetic storm index is relatively smooth on the reference day; (2) the change of geomagnetic storm index is relatively stable on the reference day, i.e., the Kp index of each period should be less than 4 and the minimum value of the Dst index should be greater than −30 nT; (3) try to select the day close to the geomagnetic storm day as the reference day, so as to avoid the influences of the GNSS receiver due to the difference of the external environment or the large differences of the distribution of the visible satellites. In this paper, the day before the geomagnetic storm (11 May 2021) is selected as the reference day, the maximum value of Kp index is 3, and the minimum offset value of Dst index is 2 nT. Figure 1 shows the time series of Kp and Dst indices on 11 and 12 May 2021.

**Figure 2:**
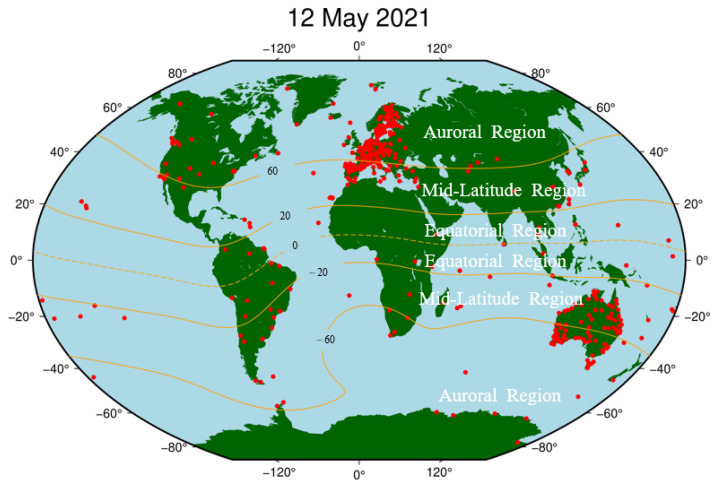
Location distribution of BDS sites on the geomagnetic storm day.

The GNSS raw observation data in the RINEX3 format utilized in this paper is provided by the IGS, UNAVCO, SOPAC, EUREF, etc., in which the number of sites containing BDS raw observation data is 491, which can cover all regions of the world and its location distribution is shown in Figure 2. In addition, the IGS precision ephemeris files, satellite and station clock solutions, antenna correction files, and tidal correction files provided by the MGEX data center are used to eliminate or reduce the PPP solution errors [29].

## 3. Definition of GNSS Observation Signal Quality Evaluation Index

### 3.1. Index Definition

(1) Calculation of lost satellite signals at a single station

In order to characterize the number of lost GNSS satellite signals during geomagnetic storms, we defined two indices, i.e., the number of lost GPS signals at a single site (SLGS) and the global average rate of lost GPS signals (GLGS), in our previous studies. We note that one or several satellite PRN numbers did not record at an epoch or a certain interval, but the corresponding observations exist before and after the loss epoch or interval, which means that the signal is lost and seriously affected. For the tri-band BDS signals, a satellite is considered out of loss if the carrier phase observations in two or more bands are 0 or blank at the corresponding epoch. For dual-frequency BDS signals, the satellite is considered to be out of lock if the carrier phase observations in one band or two bands are 0 or blank at the corresponding epoch. We first calculated the difference between the number of actual observation satellites and the number of theoretical observation satellites in a single station using Equation (1), then calculated the percentage of lost satellite signals at a single site using Equation (2).

(1) $$N_{SLGS}=\left|N_{A}-N_{T}\right|$$

(2) $$P_{SLGS}=\frac{N_{SLGS}}{N_{T}}\times 100\%\;$$

where $N_{A}$ denotes the number of actual observation satellites at a single station, $N_{T}$ denotes the number of theoretical observation satellites at a single station, that is, the number of satellites that should be observed at each epoch at each site calculated from the broadcast or precise (SP3) ephemeris files. $N_{SLGS}$ denotes the number of lost GNSS signals at a single station. *P_SLGS_* is the percentage of lost satellite signals at a single site.

Our previous studies show that the SLGS and GLGS responded well to the change of Dst. The peak value of GLGS and the standard deviation of SLGS are exponentially correlated with the magnitude of Dst and the fitting coefficient is better than 0.9. For a more detailed description of SLGS, readers are referred to [24,25]. The number of lost satellite signals at a single GNSS station under each epoch is recorded as *N_SLGS_*.

(2) Calculation of loss of lock indicator (LLI) at a single station

In the raw RINEX file, for phase observation, the Loss of Lock Indicator (LLI) ranges from 0 to 7. Number 0 or blank indicate OK or unknown, respectively. Number 1 indicates that the phase may have half-cycle ambiguity or cycle slip possible, or half-cycle of observation that cannot be handled by software. Number 2 indicates that the GNSS BOC-tracking method processes its MBOC-modulated signal (may suffer from increased noise). For tri-band BDS signals, if the carrier phase observations in two or more bands are not 0 or blank at an epoch, which means there is at least two or more observation signals at the corresponding epoch, and if at the same time, the LLI is not 0 or blank at the corresponding epoch in two or more bands, such a satellite is recorded as *N_LLI_*. Similarly, for dual-band BDS signals, if the carrier phase observations in two bands are not 0 or blank at an epoch, whilst the corresponding LLI is not 0 or blank in any of the two bands, such a satellite is also recorded as *N_LLI_*. The percentage of *N_LLI_* ($P_{LLI}$) at a single GNSS receiver is calculated using Equation (3) as follows.

(3) $$P_{LLI}=\frac{N_{LLI}}{N_{T}}\times 100\%\;$$

(3) Calculation of anomaly satellite signals at a single station

During the quiet period of geomagnetic activity, the ionospheric electron density varies relatively gently with space and time. If the carrier phase observations in two or more frequency bands are not out of loss, the difference between the product of the dual-frequency carrier phase observations and wavelengths tends to be a smooth curve. However, under the influence of geomagnetic storms, irregular changes in satellite carrier phase observations, which are affected by the inhomogeneous structure of the ionosphere, could change this regularity [30]. Following the “phase slip” idea from Afraimovich et al. [13,14], the dual-frequency carrier phase observation combination calculated by Equation (4), that is the Geometry Free (GF), is used for the detection of anomaly satellite signals affected by ionospheric irregularities.

(4) $$W_{GF}=\lambda_{1}\phi_{1}-\lambda_{2}\phi_{2}=\frac{c}{f_{1}}\phi_{1}-\frac{c}{f_{2}}\phi_{2}\;$$

The adjacent $W_{GF}$ satisfies Equation (5), anomaly satellite signals occur. We further calculated the percentage of anomaly satellite signals of a single GNSS station using Equation (6).

(5) $$|W_{GF}\left(i\right)-W_{GF}\left(i-1\right)|>m\;$$

(6) $$P_{GF}=\frac{N_{GF}}{N_{T}}\times 100\%$$

where $W_{GF}$ is the dual-frequency carrier phase observations combination. $\phi_{1}$ and $\phi_{2}$ are the dual-frequency carrier phase observations of BDS. $\lambda_{1}$ and $\lambda_{2}$ are the wavelengths of BDS satellite signals. $f_{1}$ and $f_{2}$ are the frequencies of BDS satellite signals. c is the speed of light (c=299792458 m/s). *m* is the threshold to select out anomaly satellites [31]. The absolute value less than or equal to the threshold of *m* means that there is no anomaly satellite, otherwise the satellite is determined to be an anomaly one with an abnormal signal. The number of anomaly satellite signals of a single GNSS station is recorded as *N_GF_*. The percentage of anomaly satellite signals of a single GNSS station is recorded as *P_GF_*.

(4) Definition of GNSS observation signal quality evaluation index

Considering the differences in intensity, spatial extent, and duration of ionospheric disturbances caused by different levels of geomagnetic storms, in order to make the GNSS observation signal quality evaluation index applicable to different levels of geomagnetic storms from weak to moderate to great types, the new index combines the percentage of lost satellite signals ($P_{SLGS}$), the percentage of LLI ($P_{LLI}$), and the percentage of anomaly satellite signals ($P_{GF}$), to complement each other. The percentage of affected satellites (PAS) index is defined and calculated using Equation (7) as follows.

(7) $$PAS=\frac{k_{1}\times \sum_{n=1}^{N_{S}}P_{SLGS}+k_{2}\times \sum_{n=1}^{N_{S}}P_{LLI}+k_{3}\times \sum_{n=1}^{N_{S}}P_{GF}}{N_{S}}\;$$

where the coefficients of *k*_1_, *k*_2_, and *k*_3_ are given in Table 1. *N_S_* is the number of affected BDS stations.

### 3.2. Data Processing

**Figure 3:**
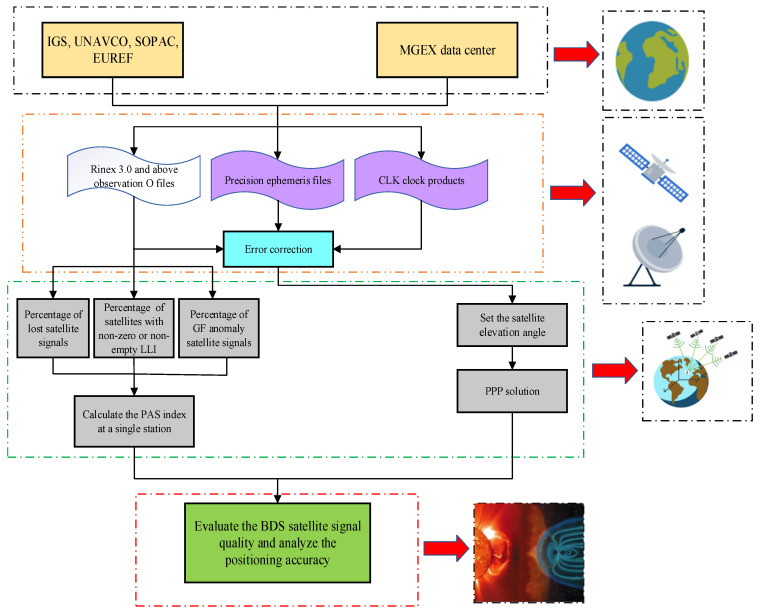
Data processing flow for BDS satellite signal quality evaluation and positioning accuracy analysis.

First, the original GNSS observation files containing the BDS signals are downloaded from the websites of IGS, UNAVCO, SOPAC, EUREF, etc., and the corresponding precision ephemeris files and clock products are obtained from the MGEX data center. Then, the percentage of lost satellite signals ($P_{SLGS}$), the percentage of LLI ($P_{LLI}$), and the percentage of anomaly satellite signals ($P_{GF}$) are utilized to calculate PAS based on the observations of a single station. On this basis, the PPP positioning solution of the abnormal station is performed, and the error analysis is conducted. Finally, the observed signal quality of global BDS during geomagnetic storms is evaluated and then its influence on positioning accuracy is analyzed, and the relationship models between the PAS index and the geomagnetic storm indices Kp and Dst are established. The complete data processing flow is shown in Figure 3.

## 4. Results

During geomagnetic storms, anomaly satellite signals detection using the GF combination for BDS observations requires the selection of appropriate thresholds. Since the BDS heterogeneous constellation includes Geosynchronous Earth Orbit (GEO) satellites, Inclined Geosynchronous Satellite Orbit (IGSO) satellites, and Middle Earth Orbit (MEO) satellites, using the same threshold for different types of satellites will result in missing or misjudging the number of anomaly satellite signals. It is recommended that the detection threshold could be selected between 0.01 and 0.02 m during moderate geomagnetic storms [32,33,34,35,36]. Then, we calculated the PAS index for all the BDS stations globally.

**Figure 4:**
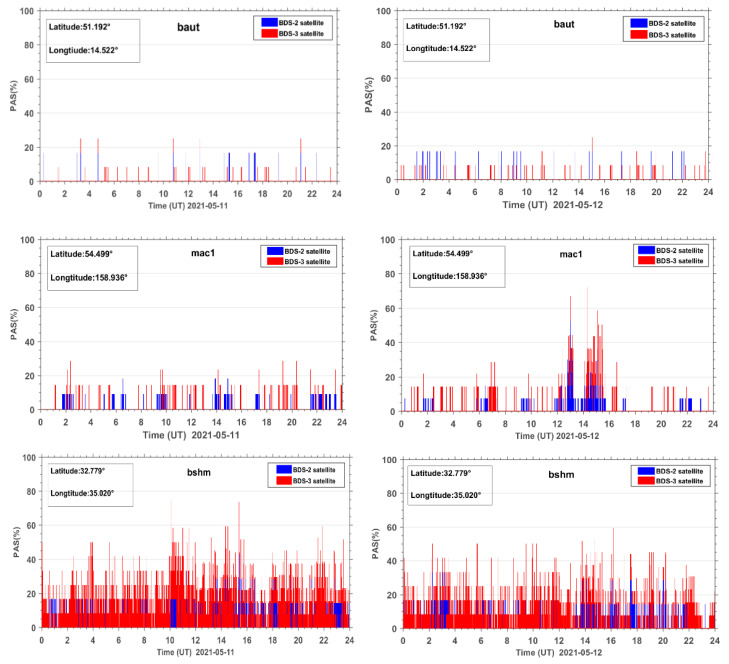
PAS time series at three BDS stations, that is baut, mac1, and bshm, during the reference day (11 May 2021) and the geomagnetic storm day (12 May 2021).

Figure 4 shows the PAS time series during the reference day and geomagnetic storm day (taking the BDS sites baut, mac1, and bshm as examples, other BDS sites are similar). As seen from Figure 4, the PAS index of the site baut does not fluctuate much during the reference day and the geomagnetic storm day. The PAS index generally fluctuates in the range from 0 to 30% and PAS change is relatively stable. For the mac1 station, during the reference day, the PAS index did not change much, similar to the site baut and there was no obvious abnormal change. On the geomagnetic storm day, the PAS index of site mac1 showed significant anomaly changes with the rapid decrease of the geomagnetic index Dst and the steep increase of the geomagnetic index Kp, and reached the first peak value of 70% during the main phase of the geomagnetic storm. The sites similar to mac1 are defined as anomaly sites affected by geomagnetic storms. The abnormal changes of PAS of site bshm both during the reference day and the geomagnetic storm day may be caused by the external observation environment or the poor performance of the GNSS receiver itself. For this reason, the sites with abnormal PAS on the reference day are called noisy sites. All noisy sites need to be identified and eliminated first before the subsequent processing. In this experiment, a total of 45 noisy sites were removed.

**Figure 5:**
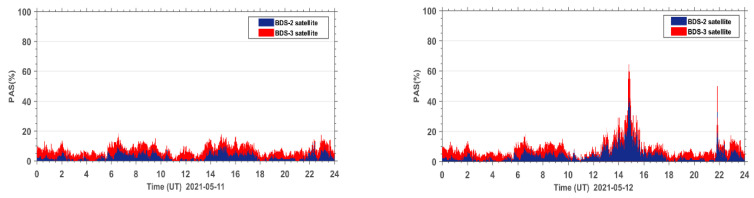
Time series of PAS for BDS sites worldwide during the reference day (11 May 2021) and the geomagnetic storm day (12 May 2021).

The PAS index of all affected sites during the reference day on 11 May 2021 and the geomagnetic storm day on 12 May 2021 are accumulated under the same epoch. The PAS time series of the global BDS sites are shown in Figure 5. It can be seen that both BeiDou 2 (BDS2) and BeiDou 3 (BDS3) systems show two anomaly peaks in PAS on the geomagnetic storm day compared to the reference day, corresponding to 15:00 UT and 22:00 UT, indicating that the geomagnetic storm has a significant impact on the satellite signals of both BDS2 and BDS3 systems. The BDS2 and BDS3 are regional and global systems constructed and developed by China. The BDS2 regional satellite navigation system refers to the second generation of the BDS satellite navigation system, which currently has 15 satellites (five GEO satellites, seven IGSO satellites, and three MEO satellites) operating in orbit. The BDS3 global satellite navigation system refers to the third generation of BDS satellite navigation system, with 29 satellites (two GEO satellites, three IGSO satellites, and 24 MEO satellites) currently operating in orbit. Although BDS3 has almost twice the number of satellites as BDS2 on 12 May 2021, we can see that the number of affected BDS3 satellite signals only has a few more affected signals than BDS2. This indicates that the BDS3 system performs much better than the BDS2 system due to BDS3 using an improved satellite signal design.

**Figure 6:**
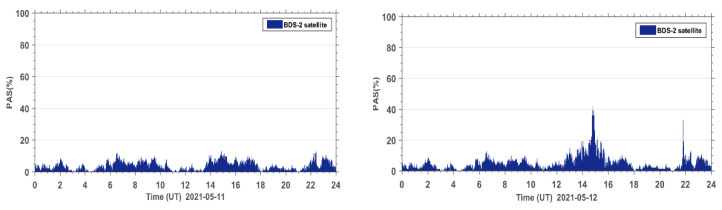
Time series of PAS for BDS2 sites worldwide during the reference day (11 May 2021) and the geomagnetic storm day (12 May 2021).

**Figure 7:**
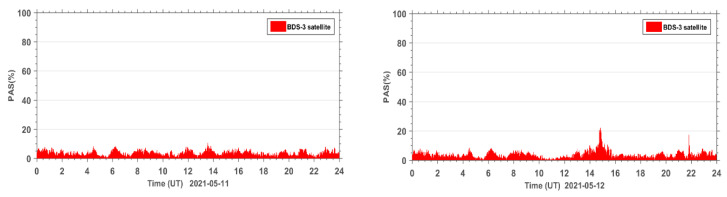
Time series of PAS for BDS3 sites worldwide during the reference day (11 May 2021) and the geomagnetic storm day (12 May 2021).

In order to further analyze the quality of the carrier phase observation signals during the geomagnetic storm for the BDS2 and BDS3 systems, the PAS index of BDS2 and BDS3 at the same sites during the reference day and the geomagnetic storm day were statistically analyzed. The PAS index of BDS2 and BDS3 at all affected sites were summed up and the time series of PAS were plotted for the global BDS2 site (Figure 6) and the global BDS3 site (Figure 7), respectively. From the statistical results in Figure 6 and Figure 7, we can see that the carrier phase observation signal quality of both BDS2 and BDS3 is susceptible to the geomagnetic storm.

**Figure 8:**
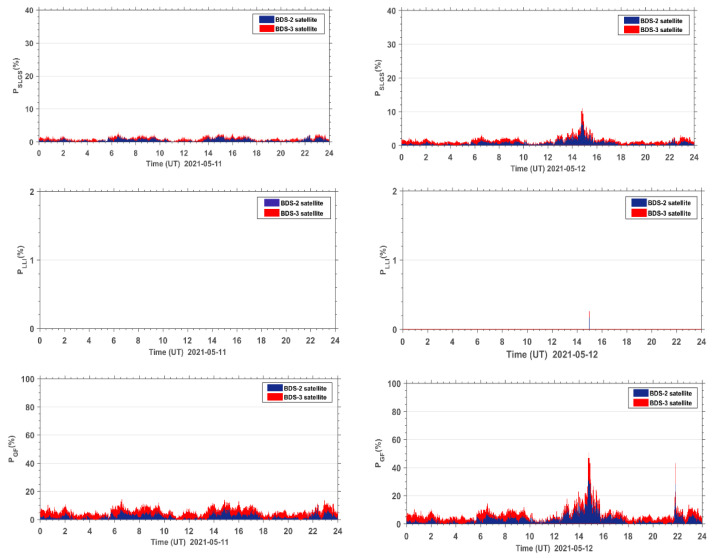
Time series of P_SLGS_, P_LLI_, and P_GF_ for BDS sites worldwide during the reference day (11 May 2021) and the geomagnetic storm day (12 May 2021).

The new PAS index is the sum of three indices, that is P_SLGS_, P_LLI_, and P_GF_. To demonstrate why the three indices should be added together to form a new index, we present the individual result of P_SLGS_, P_LLI_, and P_GF_ during the reference day and the geomagnetic storm day in Figure 8. The results show that all three indices contribute to the PAS during geomagnetic storms. The P_GF_ contributes the most to the PAS, followed by the P_SLGS_. In the case of existence of lost satellite signals, only the P_SLGS_ index can be utilized to indicate the geomagnetic storms. The P_LLI_ and P_GF_ indices, same as ROTI, would fail. When the satellite signal is not lost, the P_LLI_ index indicates the percentage of anomaly satellite signals caused by receiver performance due to geomagnetic storms, while the P_GF_ index indicates the percentage of anomaly satellite signals suffering TEC fluctuation due to geomagnetic storms with no anomalies in receiver performance. The three indices can complement each other for geomagnetic storm indication.

## 5. Discussion

### 5.1. Temporal Correlation between the Geomagnetic Storm Index and the PAS Index

**Figure 9:**
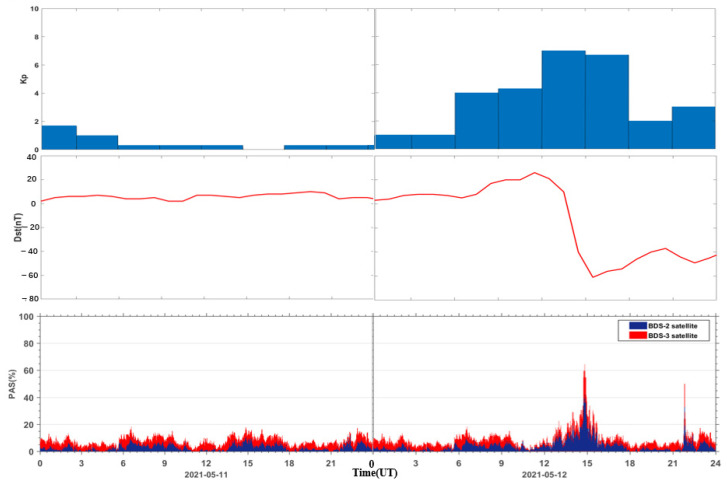
Relationship between the PAS index of global BDS sites affected by the geomagnetic storm and the geomagnetic indices Kp, Dst.

To analyze the temporal correlation between the PAS index of global BDS sites and the geomagnetic storm indices, we compared the PAS of global BDS sites during the reference day and the geomagnetic storm day with the geomagnetic indices Kp, Dst, as shown in Figure 9. It can be seen that the geomagnetic index Kp, Dst does not change much during the reference day, and the PAS time series also change relatively smoothly. Accompanied by the sharp increase of the geomagnetic index Kp and the sharp decrease of the geomagnetic index Dst during the geomagnetic storm day, the PAS index of global BDS sites affected by the geomagnetic storm show significant anomaly changes. We found that the anomaly changes of the PAS index mainly occur in the main phase and the early recovery phase of the geomagnetic storm. Along with the increase of Kp index and decrease of Dst index, the PAS index of global BDS sites affected by the geomagnetic storm gradually stabilize and return to the level of the reference day in the middle and late recovery phase.

To further quantitatively analyze the correlation between the PAS index of global BDS sites and the geomagnetic indices Kp and Dst during the geomagnetic storm day, the PAS index of global BDS sites affected by the geomagnetic storm was calculated in different time intervals. In calculating the correlation between the Kp index and the PAS index, we used the 3-h average of PAS corresponding to the time window of Kp, and the 1-h average of PAS was used to calculate the correlation between the Dst index and the PAS index. The correlation coefficients between geomagnetic indices Kp and Dst and the PAS index of global BDS sites during the geomagnetic storm day was calculated using the following equation.

(8) $$R^{2}=\frac{\sum_{i=1}^{n}\left(X_{i}-\bar{X}\right)\left(Y_{i}-\bar{Y}\right)}{\sqrt{\sum_{i=1}^{n}{\left(X_{i}-\bar{X}\right)}^{2}}\sqrt{\sum_{i=1}^{n}{\left(Y_{i}-\bar{\bar{Y}}\right)}^{2}}}\;$$

where $\;X_{i}$ represents the Kp index or Dst index at the corresponding epoch and $Y_{i}$ represents the 3-h or 1-h average PAS corresponding to the Kp index or Dst index. $\bar{X}$ and $\bar{Y}$ denote their average values, respectively. Correlation coefficient $R^{2}$ ranges from −1 to +1, and when R = 0, it indicates no correlation between the two datasets; when R < 0, it means that the two groups of data are negatively correlated; and when R > 0, it means that the two sets of data are positively correlated.

**Figure 10:**
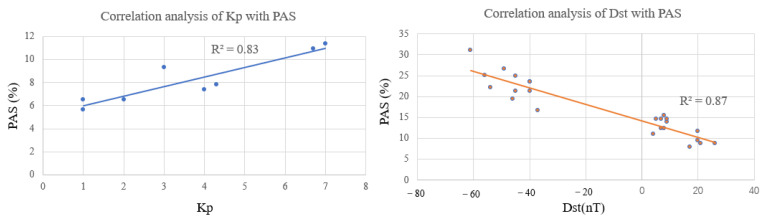
Correlation analysis between the PAS index and the geomagnetic indices Kp, Dst.

The correlation between the geomagnetic indices Kp, Dst, and the PAS index during the geomagnetic storm day is shown in Figure 10. It can be seen that the correlation coefficient between the Kp index and the PAS index during the geomagnetic storm day is 0.83 and the correlation coefficient between the Dst index and the PAS index is 0.87. It means that the geomagnetic index Kp has a good positive correlation with the change of the PAS index and a good negative correlation with the Dst index.

### 5.2. Spatial Distribution of the Affected BDS Sites during the Geomagnetic Storm

**Figure 11:**
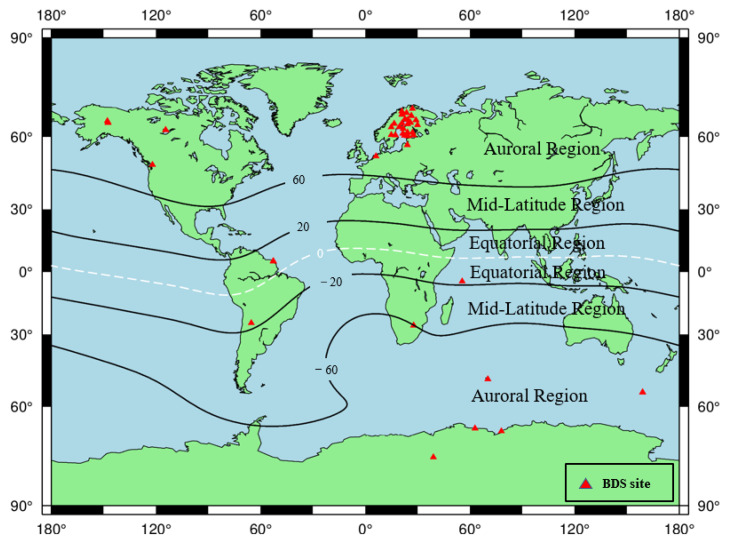
Spatial distribution map of BDS sites affected by the geomagnetic storm.

Except for the 45 noisy stations, the PAS of all the remaining 446 BDS sites during the reference day and the geomagnetic storm day was analyzed, and the sites with PAS greater than 30% during the geomagnetic storm day are considered as PAS anomaly sites. The global distribution of BDS sites with anomaly PAS affected by the geomagnetic storm is shown in Figure 11.

As seen in Figure 11, we found that most of the PAS anomaly sites are concentrated in western Europe, and sporadic BDS sites are distributed near North America, South America, Africa, and Antarctica. According to the latitudes of the geomagnetic field, we also found that the BDS sites affected by the geomagnetic storm are mainly located in auroral regions and equatorial regions of the geomagnetic field. The number of sites in auroral regions is significantly higher than those in equatorial regions. For the polar regions, because the polar ionospheres are the key regions in the solar-terrestrial energy coupling process, and various dynamical processes caused by the solar wind-magnetosphere interaction can directly affect the polar ionosphere, causing numerous plasma processes in the polar ionosphere such as storm-enhanced density, the tongue of ionization, polar cap patches, auroral blobs, and auroral precipitation might have serious effects on BDS signals (e.g., [37,38,39]). For the equatorial region, the classic “fountain effect” is responsible for the Appleton anomaly, thus the ionospheric electron density in the geomagnetic equatorial regions is higher than the middle latitude regions. In addition, the ionospheric plasma bubbles or plumes, resulting from the collisional plasma Rayleigh–Taylor instability, could produce smaller-scale ionospheric irregularities responsible for both amplitude and phase scintillations and/or loss of satellite signals in L-band (e.g., [12,40]).

### 5.3. Comparison between the PAS Index and the ROTI Index

**Figure 12:**
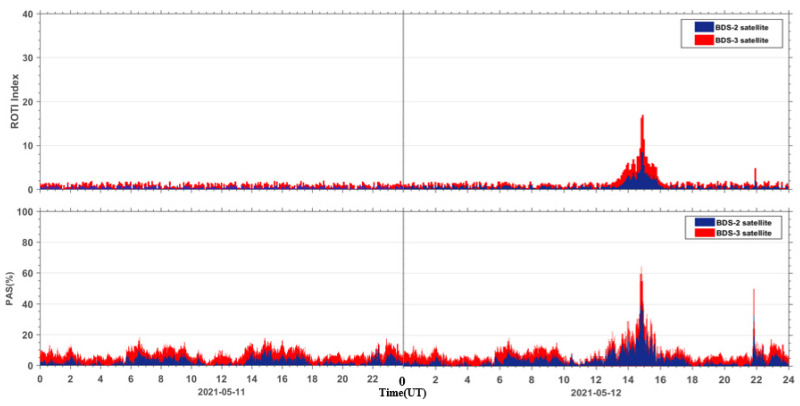
The comparison results of the ROTI index and the PAS index.

**Figure 13:**
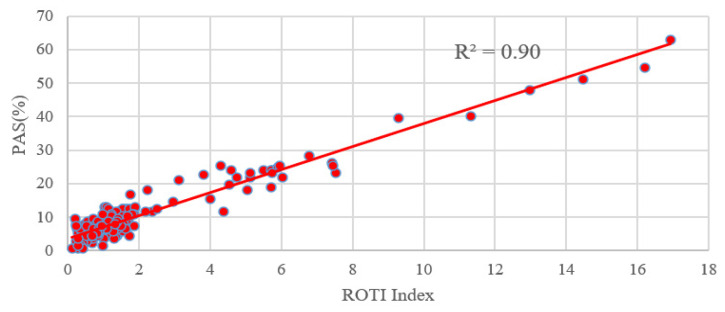
Correlation analysis between the ROTI index and the PAS index.

In order to compare the PAS index with the ROTI index, we performed a statistical calculation on the ROTI index for the same stations around the world. From Figure 12, we can see that both the ROTI index and the PAS index are indicative to this moderate geomagnetic storm. We also calculated the correlation between the ROTI index and the PAS index during the geomagnetic storm day. Due to the different sampling intervals for the two indices, the PAS index of each station was extracted at a 5-min interval, same as the ROTI index. As seen in Figure 13, the PAS index has a good correlation with the ROTI index with a correlation coefficient of 0.9.

**Figure 14:**
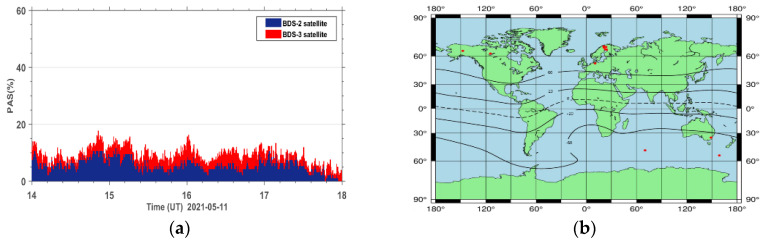
(**a**) The PAS index during the period of 14–18 h on 11 May 2021 and (**b**) the global distribution map of BDS sites affected by ionospheric irregularities in the geomagnetic auroral regions.

The high correlation between the PAS index and the ROTI index demonstrates that the proposed index is as good as the ROTI index. Furthermore, there are two more advantages for the new PAS index. The first one is that the PAS index has the ability to indicate the percentage of lost satellite signals (P_SLGS_) and the percentage of loss of lock indicator (P_LLI_), which is not taken into account in the ROTI index. The second one is that the ROTI index suppresses the ionospheric effect on the BDS signals during both the quiet and disturbed periods as seen in the upper portion of Figure 12. We take a period of 14–18 h on 11 May 2021 as an example to show the differences between the two indices. We can see that the ROTI shows no signals during this period in Figure 12. We zoomed in the PAS index and extracted the BDS sites that contributed to the PAS index during the same period. We also plotted the global distribution map of these BDS sites, as seen in Figure 14. We can see that these affected sites are mainly located in the geomagnetic high-latitude regions. This might indicate that the PAS index also has the ability to indicate the ionospheric effect on the BDS sites during quiet periods. This is because the ionosphere is active in the geomagnetic high-latitude regions even though the geomagnetic activity is quiet. We will further study this effect in future work. Overall, we think that the PAS index is a more comprehensive index than the ROTI index to quantitatively evaluate the quality of the carrier phase observation signals of BDS during geomagnetic storms.

### 5.4. Effect of the Geomagnetic Storm on Precision Point Positioning (PPP)

**Figure 15:**
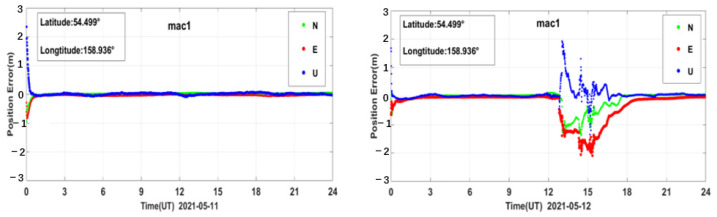
PPP error time series of BDS station mac1 during the reference day and the geomagnetic storm day.

PPP generally refers to a high-precision positioning technique using a dual-frequency GNSS receiver with pseudo-range observations, carrier phase observations, and products such as precision ephemeris and satellite clock solutions provided by some organizations, such as IGS [41,42,43]. In this paper, the PPP solution was performed on the BDS data for all the sites around the world during the reference day and the geomagnetic storm day [44,45]. We used Net-Diff software for PPP calculation, which is developed based on RTKLIB software [46]. In the solving process, we only used the BDS observations to perform PPP calculation. Its solution strategy adopts the PPP kinematic positioning model, which eliminates the influence of the ionospheric first-order term through the dual-frequency ionosphere-free combination; the PPP solution is performed after modeling and correcting for the errors related to the troposphere, phase wind-up, and antenna phase center deviation, and the positioning accuracy can reach the centimeter level, or even millimeter level [47,48]. The signal bands used in the single station PPP solution are the same as the bands used in the single station PAS calculation. The PPP error results of all the above-mentioned PAS anomaly BDS sites were analyzed and compared during the reference day and the geomagnetic storm day. We take the site mac1 as an example; the PPP error time series during the reference day and the geomagnetic storm day are shown in Figure 15, other sites are similar.

As shown in Figure 15, we can see that the PPP of BDS is affected by the geomagnetic storm significantly. The PAS index and the PPP errors did not change much on 11 May. However, during the main phase and the recovery phase of the geomagnetic storm on 12 May the PAS index and the PPP errors showed a significant abnormality, which might be caused by the geomagnetic storm.

To statistically analyze the PPP errors with the PAS index, we calculated the horizontal RMSE (*H_RMSE_*) as the square root of the sum of the squares of the X-direction RMSE and the Y-direction RMSE of a single BDS station. The vertical RMSE (*V_RMSE_*) is the RMSE of the Z-direction of a single BDS station.

(9) $$H_{RMSE}=\sqrt{X_{RMSE}^{2}+Y_{RMSE}^{2}}\;$$

where *X_RMSE_* is the root mean square error in the *X* direction of the BDS site, *Y_RMSE_* is the root mean square error in the *Y* direction of the BDS site.

**Figure 16:**
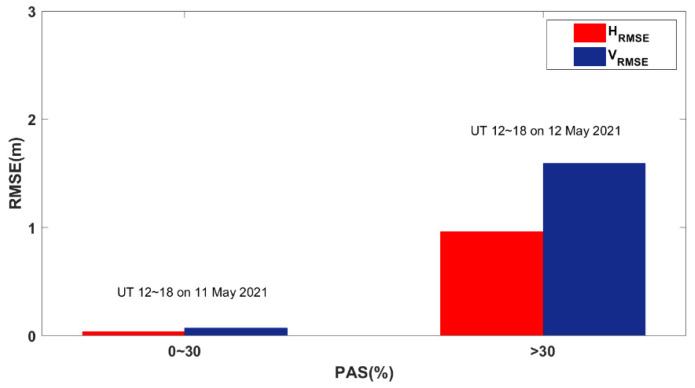
The PAS index range corresponds to the mean horizontal and vertical error of PPP.

Figure 16 shows the average PPP errors from 12:00 UT to 18:00 UT in the horizontal and vertical directions for all the affected BDS sites worldwide during the reference day and the geomagnetic storm day, respectively. As the PAS index varies from 0 to 30% on the reference day, the corresponding mean value of *H_RMSE_* is 0.036 m and the mean value of *V_RMSE_* is 0.071 m. As the PAS index increases on the geomagnetic storm day, the mean value of *H_RMSE_* and *V_RMSE_* increases significantly. The *H_RMSE_* and *V_RMSE_* are 0.933 m and 1.592 m on average.

The satellite elevation cutoff angle is the angle between the observed satellite’s emitted ray to the site and the horizontal plane. The number of satellites received by BDS stations can be effectively controlled by the elevation angle [49]. In order to avoid the impact of errors such as the multipath effect and atmospheric delay on the global BDS positioning error, the number of satellites can be eliminated by setting the satellite elevation cutoff angle to control the number of satellites. In this paper, we analyzed the PPP errors of global BDS with different satellite elevation cutoff angles during the geomagnetic storm day.

**Figure 17:**
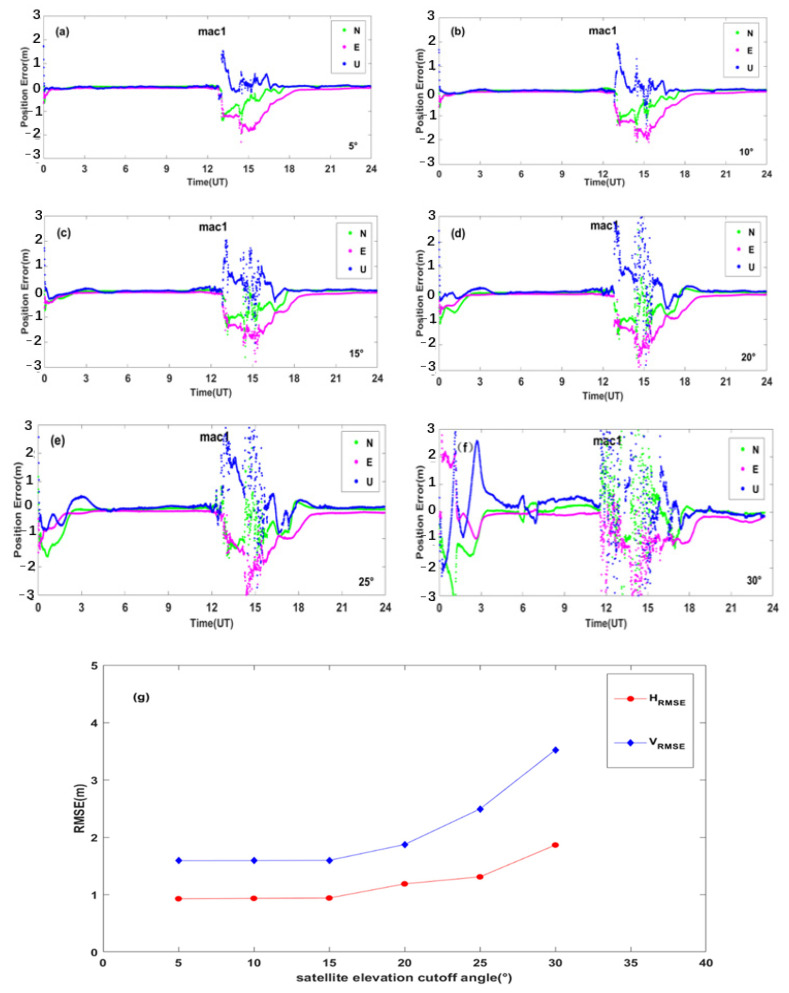
(**a**–**f**) Positioning error at station mac1 for different satellite elevation cutoff angles; (**g**) the dependence of PPP positioning errors on different elevation cutoff angles.

Figure 17a–f shows positioning errors of BDS station mac1 at different satellite cutoff elevation angles. It can be seen that the positioning errors vary with satellite cutoff elevation angles. We further calculated the average *H_RMSE_* and *V_RMSE_* for all the BDS sites affected by geomagnetic storms globally at different satellite elevation cutoff angles. Figure 17g shows the dependence of PPP positioning errors on different elevation cutoff angles. It can be seen that the average *H_RMSE_* and *V_RMSE_* do not vary much when the satellite elevation cutoff angle of 5°, 10°, and 15°. When the satellite elevation cutoff angle is equal to or larger than 20°, the average *V_RMSE_* increases steeply. Therefore, we suggest that the satellite cutoff elevation angle could be selected less than or equal to 15° for PPP positioning during geomagnetic storm days.

## 6. Conclusions

Taking a moderate geomagnetic storm that occurred on 12 May 2021 as an example, the statistical evaluation of the signal quality observed at global BDS sites was carried out and the PPP error was analyzed. The comparative analysis of the observational signal quality of BDS2 and BDS3 yields the following main conclusions:

(1) By combining the percentage of lost satellite signals, the percentage of the Loss of Lock Indicator (LLI) not 0 or blank and the percentage of anomaly satellite signals detected by GF at each epoch of a single station, a new index PAS was defined to evaluate the signal quality of BDS system. The results show that the PAS index has a strong correlation with both geomagnetic indices Kp and Dst, with a good positive correlation with Kp and a good negative correlation with Dst. It indicates that the PAS index can effectively evaluate the quality of BDS observation signals during moderate geomagnetic storms and avoid the shortcomings of the ROTI index due to the effect of lost satellite signals.

(2) The PAS of BDS2 and BDS3 at various stations around the world were analyzed separately. The results show that the carrier phase observation signal quality of BDS2 and BDS3 satellites are both significantly affected during this moderate geomagnetic storm. We will further statistically analyze the geomagnetic storm effect on BDS2 and BDS3 satellite signals for more cases in future work.

(3) The PAS anomalies and the PPP positioning error anomalies caused by the moderate geomagnetic storm are well correlated. The positioning error anomalies are mainly concentrated in the main phase and early recovery phase of the geomagnetic storms. The affected stations are mainly distributed in the geomagnetic high- and low-latitude regions. The number of stations in the high-latitude region is higher than those in the low-latitude region.

(4) In the process of PPP positioning of BDS during the moderate geomagnetic storm, the influence of the elevation angle of the satellite on the PPP error should be considered adequately. It is recommended to set the elevation cutoff angle at less than or equal to 15°.

## Figures and Tables

**Table 1:** The coefficients for PAS calculation.

Satellite Signal Status		Coefficients		
		k1	k2	k3
Satellite signal loss		1	0	0
Satellite signal exists	LLI = 1~7	0	1	0
	LLI = 0 or Blank	0	0	1

## Data Availability

The data presented in this study are available on request from the corresponding author. The data are not publicly available due to privacy issues.
